# Analysis of clinical features and prognostic factors in patients with hepatic hydrothorax: a single-center study from China

**DOI:** 10.1186/s12876-022-02412-9

**Published:** 2022-07-07

**Authors:** Bo Ma, Tianling Shang, Jianjie Huang, Zhixin Tu, Yan Wang, Yujin Han, Xiaoyu Wen, Qinglong Jin

**Affiliations:** 1 Department of Hepatology, The First Hospital of Jilin University, No. 71 Xinmin Street, Changchun, 130000 Jilin Province China; 2 Department of Neurology, The First Hospital of Jilin University, No. 71 Xinmin Street, Changchun, 130000 Jilin Province China; 3 Department of Hepatology, The First Hospital of Jilin University, No. 1, Xinmin Street, Chaoyang District, Changchun, 130021 Jilin China

**Keywords:** Hepatic hydrothorax, Clinical features, Decompensated cirrhosis, Portal hypertension, Prognostic factors

## Abstract

**Background:**

The clinical features and factors affecting the prognostic survival of hepatic hydrothorax (HH) are currently unknown.

**Methods:**

We conducted a retrospective cohort study of 131 patients with HH using the Kaplan–Meier method and Cox proportional hazards regression analysis to assess factors influencing the prognosis of HH.

**Results:**

A total of 131 patients were enrolled: the male to female ratio was 80:51 (1.59:1), and the mean age was 52.76 ± 11.88 years. Hepatitis B cirrhosis was the main cause of HH, and abdominal distention and dyspnea were the most common clinical signs. Ascites was present in varying amounts in all patients and was the most common decompensated complication, with pleural effusions mostly seen on the right side (107/131; 82%), followed by the left side (16/131; 12%) and bilateral effusions (8/131; 6%). For overall survival without transplantation, the estimated median survival time was 21 (95% confidence interval [CI]:18–25) months, and survival rates at 6 months, 1 year, and 2 years were 77.2%, 62.4%, and 29.7%, respectively. After controlling for covariates that were associated with liver-related mortality in the univariate analysis, males (hazard ratio [HR]: 1.721, 95% CI: 1.114–2.658, *P* = 0.005) and combined hepatic encephalopathy (HR: 2.016, 95% CI: 1.101–3.693, *P* = 0.001) were found to be associated with an increase in liver-related mortality.

**Conclusions:**

In this cohort of HH patients without liver transplantation, male sex and hepatic encephalopathy were associated with a higher risk of liver-related death.

## Introduction

Hepatic hydrothorax (HH) is a pleural effusion that is generally higher than 500 ml in size and is related to cirrhosis and portal hypertension in the absence of any cardiac, pulmonary, or pleural illness [1]. HH occurs in approximately 5-15% of cases, is a rare complication of end-stage liver disease, can lead to hypoxia, respiratory distress, and infection, predicts a poor prognosis, and occurs independently of the specific cause of cirrhosis [2, 3]. HH is more prevalent on the right side of the chest (85%), although it can also occur on the left side (13%) and bilaterally (2%), even in the absence of clinical ascites [4]. Most studies suggest that the pathophysiological pathway through which HH occurs is through the formation of peritoneal-pleural defects through microscopic and macroscopic diaphragmatic defects. These diaphragmatic abnormalities are more prevalent in the right diaphragm, which is more fibrous and prone to collagen fiber degradation, and contribute to the prevalence of right-sided pleural effusions in HH patients. Treatment of HH usually includes medical management with diuretics and sodium restriction and therapeutic thoracentesis as necessary. Dietary sodium restriction and diuretics are preferred for long-term management or mild pleural effusion, while therapeutic thoracentesis is usually used for the acute relief of symptoms. However, transjugular intrahepatic portosystemic shunts (TIPS), liver transplantation (LT), and surgical repair of diaphragmatic defects are also advocated for some patients, particularly those with refractory HH.

In recent years, HH with hepatopulmonary syndrome and pulmonary hypertension have been recognized as the main pulmonary manifestations of chronic liver disease and cirrhosis [5]. Patients with HH are more likely to have acute kidney injury, hepatic encephalopathy, infectious shock and higher mortality [2]. A recent study showed that HH is an independent decompensated event associated with long-term mortality in patients with cirrhosis [6]. Although HH usually occurs in end-stage liver disease, the prognostic impact on HH is currently unknown. In this study, we further explored the clinical characteristics and factors associated with the prognosis of patients by retrospectively analyzing 131 patients with HH admitted to our hospital.

## Materials and methods

### Study population

**Fig. 1:**
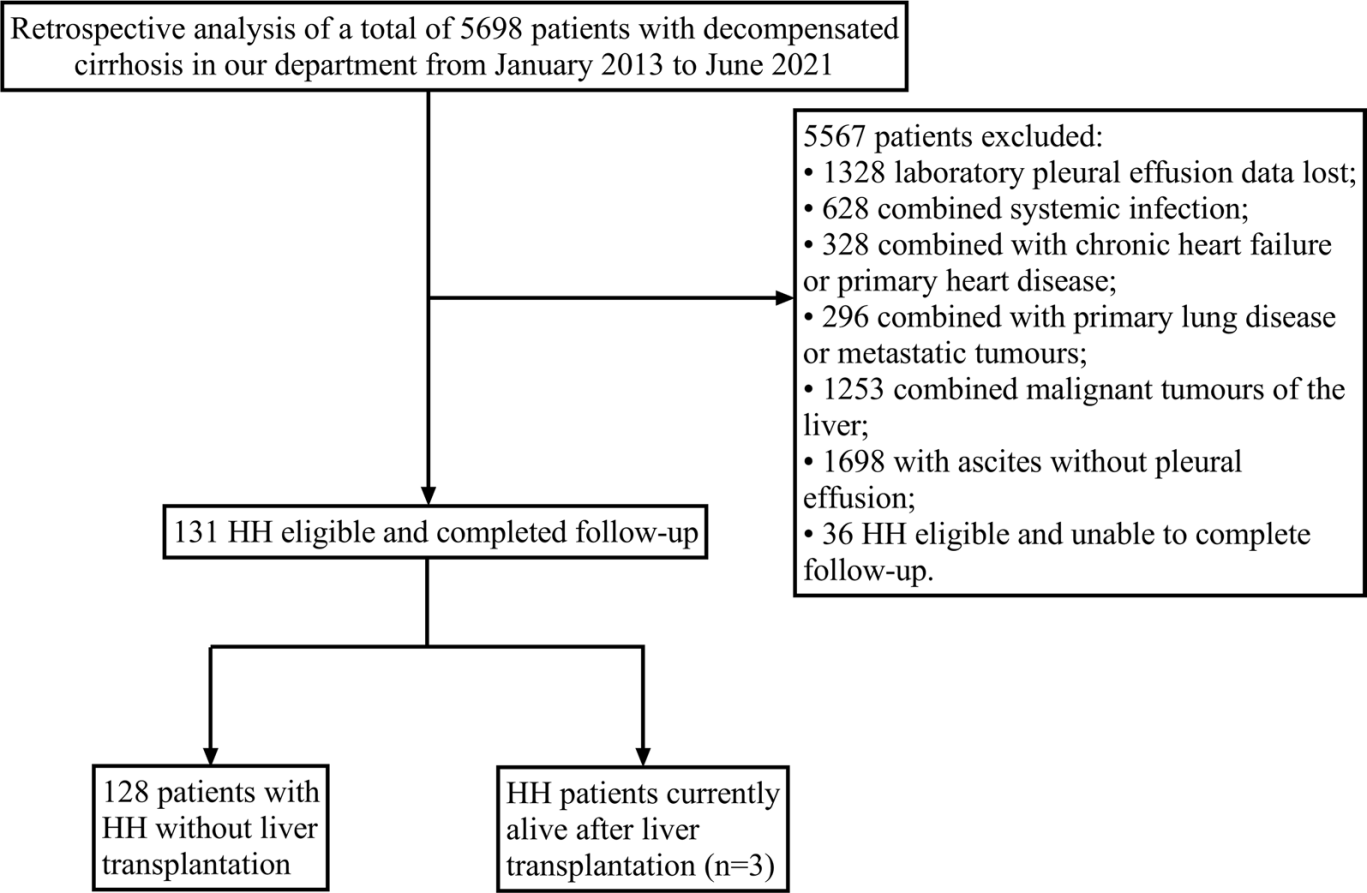
Study the flow diagram

We conducted a retrospective cohort analysis of 5698 patients diagnosed with decompensated cirrhosis from January 2013 to June 2021 at the Department of Hepatology, First Hospital of Jilin University, China. Follow-up data were collected until December 30, 2021. All relevant clinical and laboratory data at the time of first diagnostic admission, including a complete medical history, were collected. Decompensated cirrhosis and decompensating events were defined according to the latest EASL Clinical Practice Guidelines on Decompensated Cirrhosis [7]. The inclusion criteria were as follows: (1) known diagnosis of cirrhosis, either biopsy confirmed or based on clinical complications present in the clinic consistent with cirrhosis; (2) known diagnosis of pleural effusion on chest radiography or lung computed tomography (CT); (3) pleural effusion consistent with known features of hepatic pleural fluid but not considered consistent with the presence of infection, malignancy or other known chronic disease; (4) no history of primary cardiopulmonary dysfunction, including but not limited to congestive heart failure; and (5) portal hypertension as determined by esophageal varices, portal hypertensive gastropathy, ascites, portal vein thrombosis or elevated hepatic venous pressure gradient (HVPG). The criteria for the exclusion of subjects were as follows: (1) combined malignancy or major organ failure; (2) patients on hormonal or immunosuppressive agents; and (3) incomplete clinical data or information on additional tests. All patients were screened for ascites and pleural effusion using ultrasound on admission. In addition, all patients underwent standard chest radiographs or CT of the lungs to detect underlying lung disease (pneumonia, tumors, and other lesions). Patients with clinical, laboratory or electrocardiographic suspicion of heart failure underwent cardiac ultrasound to rule out decompensated heart disease. Finally, 131 adult HH patients were included in this analysis (Fig. 1). The study protocol was approved by the Ethics Committee of the First Hospital of Jilin University and was conducted according to the principles of the Declaration of Helsinki. Written general informed consent was obtained from all participants.

### Study variables

All demographic and clinical information was confirmed directly from the electronic medical record, including demographic data (age and sex), serum biochemical information (alanine aminotransferase (ALT), aspartate aminotransferase (AST), alkaline phosphatase (ALP), gamma-glutamyl transpeptidase (GGT), total bilirubin (TBIL), albumin (ALB), creatinine (Cr), serum sodium, international normalized ratio (INR), lymphocyte absolute count (LY), neutrophil absolute count (NE), neutrophil-to-lymphocyte ratio (NLR), platelet count (PLT), prothrombin time (PT), and imaging features. The diagnosis of cirrhosis was confirmed by liver biopsy or ultrasound without regard to the presence of portal hypertension. The Child–Pugh score was calculated from the original article published by Pugh et al. in 1973 [8]. The MELD-Na score was calculated from Biggins et al. [9]. The ALBI scoring and grading system was implemented as described by Johnson et al. [10]. The Child–Pugh score is based on five indicators: serum ALB, TBIL, PT, ascites and hepatic encephalopathy, with a score of 5–6 for grade A, 7–9 for grade B and 10–15 for grade C. The criteria for judging the amount of pleural effusion are as follows: If the amount of pleural fluid is greater than 6 cm or greater than the 7th rib space by ultrasound or chest radiograph, it is a large amount; If the fluid level is 3–6 cm or greater than the 8th rib space, it is a medium amount; If the fluid level is less than 3 cm or the angle of the rib diaphragm is blunt, it is a small amount. Overt encephalopathy was defined as grade 2 to 4 liver encephalopathy according to the West Haven criteria [11]. Ascites was classified according to the most recent position paper published by the International Ascites Club [12].

All blood samples were tested in the Clinical Laboratory of Jilin University’s First Hospital. An automated biochemical analyzer (7600–210, Hitachi, Japan) was used to detect blood biochemical indices. According to the manufacturer’s instructions, the total blood count was determined using a SYSMEX XN-9000 hematological analyzer (Sysmex Corporation, Kobe, Japan). Clotting tests were carried out with the automated coagulometer “SYSMEX CS-5100” utilizing the clotting technique (Sysmex Corporation, Kobe, Japan).

### Outcomes

We collected details of HH treatment, date of death, LT, and final follow-up. The primary outcome was liver-related death [death from liver failure, or hepatocellular carcinoma (HCC)] or LT. Our primary time point analysis identified the relationship between factors that may have affected prognosis and death, which was calculated from the date of first diagnosis of HH to the date of death.

### Statistical analysis

Categorical data were expressed as numbers (n) and proportions (%). Continuous variables were expressed as the means (± standard deviation) when data were normally distributed and medians (quartile 25-quartile 75) when data were not normally distributed. The Shapiro–Wilk (W test) prevailed for the normality test. The proportional hazard assumption was assessed using the Schoenfeld residuals for continuous variables and using the graphical method for categorical variables. The variables that seemed to satisfy the proportional hazard assumption were incorporated into the univariate Cox proportional hazard model to assess the effect of different variables on survival time. The contribution of each variable was estimated by the risk ratio and its 95% confidence interval. All variables with a significant effect on survival (*P* < 0.1) were included in the multivariate Cox proportional hazard model. In the multivariate Cox regression model, the variables that might be considered were further removed step by step, and the change of hazard ratio (HR) value of the target variable (the first variable) was observed after removing a variable. If the change in HR value after removal was more than 10%, the variable was ultimately retained. If not, the variable was removed. For categorical variables, the survival outcomes were compared with both methods (the Cox regression models and the Kaplan–Meier method of log-rank tests), and they used the Kaplan–Meier estimates to display survival curves. The results of the Cox proportional hazards regression analysis are presented as hazard ratios (HRs) and their corresponding 95% confidence intervals (CIs). Our analysis for identifying factors associated with mortality/survival is an exploratory analysis. All statistical analyses were performed using SPSS Statistics 26 (IBM, New York, NY, USA) and R version 4.1.3. A two-sided *P* value < 0.05 was considered statistically significant.

## Results

### Demographics

During the study period, 5698 patients with decompensated cirrhosis were screened, and according to the inclusion and exclusion criteria, follow-up data were obtained for 131 patients who met the diagnostic criteria for hepatic hydrothorax. There were more males than females, with a male to female ratio of 1.67:1 in HH patients, with 80 men (61.1%) and 51 women (38.9%). The minimum age was 25 years and the maximum age was 81 years, with a mean age of 52.76 ± 11.88 years and a median age of 58 years. At the time of primary treatment, post-hepatitis B cirrhosis was the most common cause in 55 cases (42%), followed by alcoholic cirrhosis in 29 cases (22.1%), post-hepatitis C cirrhosis in 15 cases (11.5%), primary biliary cirrhosis in 10 cases (7.6%), and other etiologies in 22 cases (16.8%) (see Table 1).

**Table 1:** Demographics, Clinical manifestations, imaging findings and complications in the decompensated phase

	No. percent of patients (%) [n = 131]
Age (year), mean ± SD	52.76 ± 11.88
Sex Male	80(61.1%)
Female	51(38.9%)
Etiology	
Hepatitis B virus(HBV)	55(42%)
Hepatitis C virus(HCV)	15(11.5%)
Alcohol	29(22.1%)
Primary biliary cirrhosis(PBC)	10(7.6%)
Other	22(16.8%)
Respiratory difficulties	89(67.9%)
Cough	30(22.9%)
Payment	54(41.2%)
Bloating	108(82.4%)
Jaundice	54(42.2%)
Liver palm	34(26%)
Spider mole	25(19.1%)
Splenomegaly	110(84%)
Location of pleural effusion	
Right side	107(81.7%)
Left side	16(12.2%)
Bilateral	8(6.1%)
Volume of pleural effusion	
Small amount	4(3.1%)
Medium amount	19(14.5%)
large amount	108 (82.4%)
Combined with massive ascites	87 (66.4%)
Compression dysplasia	96 (73.3%)
Liver Failure	21 (16%)
Hepatic encephalopathy	16 (12.2%)
Gastrointestinal bleeding	16 (12.2%)
Pleural fluid infection	30 (22.9%)
Spontaneous peritonitis	18 (13.7%)
Hyponatremia	52 (39.7%)
Renal insufficiency	24 (18.3%)
Hepatorenal syndrome	9 (6.9%)

Data are expressed as mean (± standard deviation), number (proportion)

### Clinical manifestations, imaging findings, and combined decompensated complication events

At the time of first diagnosis, most patients had multiple complaints, with the most common symptoms being abdominal distention (82.4%), difficulty breathing (67.9%), poor appetite (41.2%), and cough (22.9%). Splenomegaly (84%) was the most common sign. Only pleural effusion on the right side was found in more than three quarters of the patients (107 out of 131; 81.7%). Left-sided effusion alone was found in 16 cases (12.2%), and bilateral effusion was found in 8 cases (6.1%). According to radiological criteria, massive pleural fluid accumulation predominated in 108 cases (82.4%), followed by moderate amounts in 19 cases (14.5%) and small amounts in 4 cases (3.1%). All patients had ascites, including 87 cases (66.4%) with massive pleural effusion combined with massive ascites. Approximately 96 cases (73.3%) presented with manifestations of compressive pulmonary atelectasis. Ascites was present in varying amounts in all patients and was the most common complication of the decompensated phase. This was followed by hyponatremia in 52 cases (39.7%), pleural fluid infection identified by pleural fluid bacterial culture in 30 cases (22.9%), renal insufficiency in 24 cases (18.3%), liver failure in 21 cases (16%), peritonitis in 18 cases (13.7%), hepatic encephalopathy and gastrointestinal bleeding in 16 cases each (12.2%) and hepatorenal syndrome in 9 cases (6.9%) (see Table 1).

### Laboratory features

Routine blood collection and prognostic score data are shown in Table 2. The laboratory data were noteworthy in that most patients did not show neutrophilia, and thrombocytopenia was typical. Serum cholinesterase and serum albumin levels were generally low. Almost all patients had significant liver dysfunction, including elevated bilirubin levels, prothrombin time (PT), thromboplastin time (TT), activated partial thromboplastin time (APTT), and international normalized ratio (INR), and significantly lower than normal prothrombin activity (PTA) (58.86 [55.06–62.65] %).

**Table 2:** Laboratory Features

	At first diagnosis	Range of reference values
AST (U/L)	60.68 (50.69, 70.67)	15–40.0
ALT (U/L)	42.42 (33.89, 50.96)	9–50.0
GGT (U/L)	77.71 (62.02, 93.40)	10–60.0
ALP (U/L)	121.18 (107.48, 134.88)	45–125.0
CHE (U/L)	2288.09 (2097.47, 2478.42)	4620–11,500
ALB (g/L)	26.49 (25.62, 27.37)	40–55.0
TBIL (umol/L)	65.68 (53.68, 77.67)	0.0–26.0
DBIL (umol/L)	30.81 (24.15, 37.46)	0.0-6.8
IBIL (umol/L)	34.82 (28.94, 40.70)	5.0–20.0
Cr (umol/L)	78.72 (71.97, 85.46)	57–97
BUN (mmol/L)	7.80 (6.82, 8.77)	3.1-8.0
FBG (mmol/L)	7.22 (6.61, 7.83)	3.1–6.1
NE(10ˆ9/L)	3.77 (3.22, 4.32)	1.80–6.30
LY(10ˆ9/L)	1.00 (0.85, 1.14)	1.10–3.20
NLR	3.30 (2.17, 5.91)	0.56–5.73
PLT(10ˆ9/L)	85.49 (75.72, 95.26)	125–350
TT (s)	18.20 (17.65, 18.76)	11.0–21.0
APTT (s)	37.88 (36.30, 39.47)	21–33
PT (s)	17.22 (16.34, 18.10)	9.0–13.0
INR	1.45 (1.38, 1.52)	0.8–1.2
PTA(%)	58.86 (55.08, 62.65)	80–120

Data are expressed as mean (± standard deviation), number (proportion) and median (quartile 25, quartile 75)

*ALT* alanine aminotransferase, *AST* aspartate aminotransferase, *ALP* alkaline phosphatase, *GGT* gamma-glutamyl transpeptidase, *CHE* cholinesterase, *ALB* albumin, *TBIL* total bilirubin, *DBIL* direct bilirubin, *IBIL* indirect bilirubin, *Cr* Creatinine, *BUN* blood urea nitrogen, *FBG* fasting blood glucose, *NE* Neutrophil absolute count, *LY* Lymphocyte absolute count, *NLR* Neutrophil-to-lymphocyte ratio, *PLT* platelet, *TT* Thrombin time, *APTT* Activated partial thromboplastin time, *INR* International normalized ratio, *PT* prothrombin time, *PTA* Prothrombin activity

### Treatment and overall survival without transplantation

**Fig. 2:**
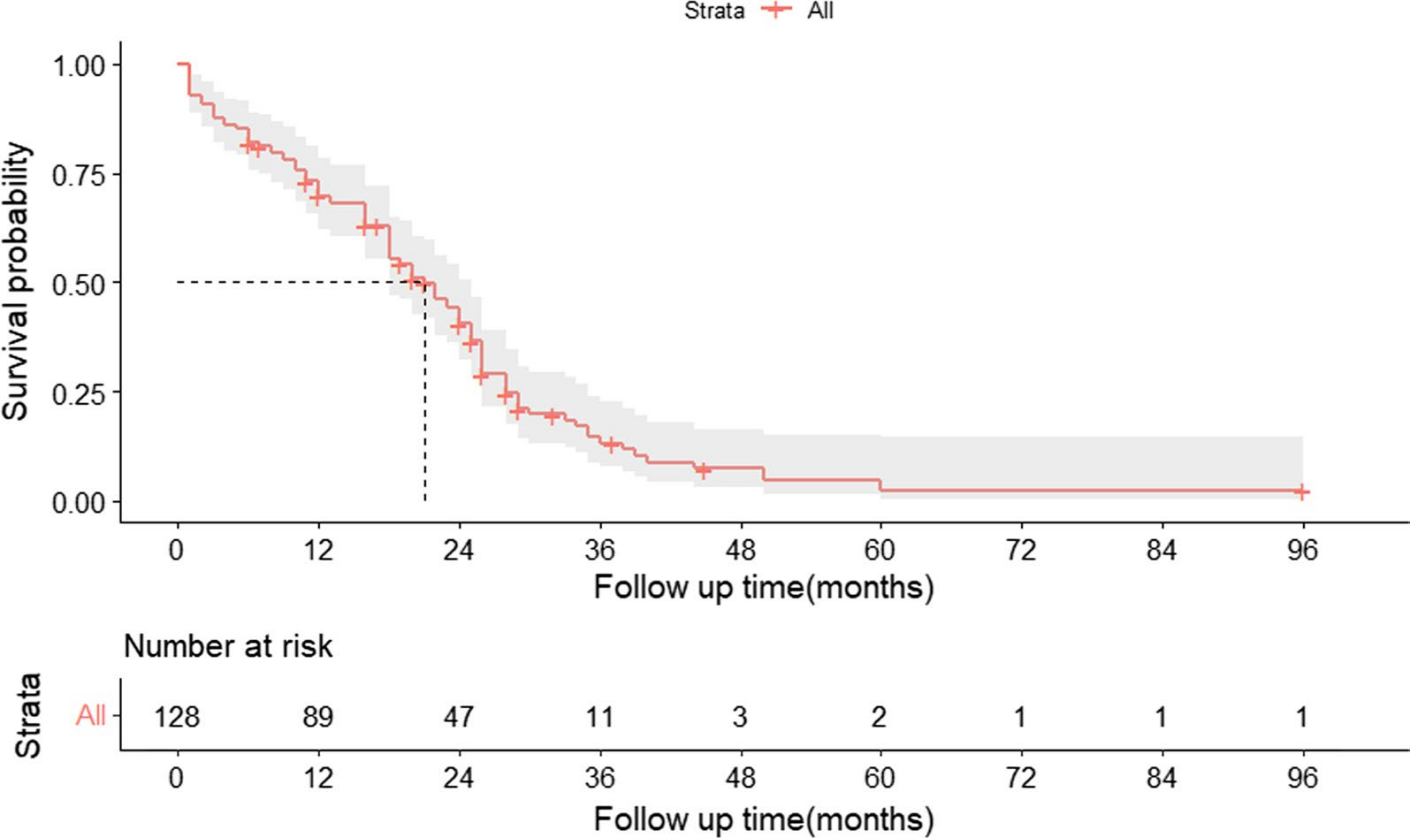
Kaplan-Meier survival curves for transplant-free overall survival in HH patients. The 95% confidence interval of the survival probability is marked by the shading. The dotted line indicates the median overall survival. Every tick mark indicates a censored patient

There were 131 patients at first diagnosis, of whom 20 (15%) were treated with drug management alone, with a mean age of 53.35 ± 11.91 years, a median MELD score of 10.14 (0.47–24.76) and an estimated median time of 19 months. A total of 111 (75%) patients were treated with medication and thoracic intubation, with a mean age of 57.32 ± 11.83 years, a median MELD score of 10.22 (0.0-30.50), and an estimated median time of 22 months. During the later stages of treatment, one patient was treated with TIPS, with a MELD score of 16 and a survival time of 32 months, and three patients were treated with liver transplantation, with a mean MELD score of 18, all of whom are currently alive (see Table 3). For overall survival without transplantation, the median follow-up time was 45 (interquartile range [IQR]: 25–96) months, the estimated median survival time was 21 (95% CI:18–25) months, and the survival rates at 6 months, 1 year, and 2 years were 77.2%, 62.4%, and 29.7%, respectively (Fig. 2).

**Table 3:** Comparison of treatment modalities

Treatment modalities	No.(%)	Age (year)	MELD Score	Survival time (months)
Drugs (At first diagnosis)	20/131(15%)	53.35 ± 11.91	10.14(0.47–24.7)	19(14–23)
Thoracic intubation (At first diagnosis)	111/131(75%)	57.32 ± 11.83	10.22(0.00-30.50)	22(17–26)
TIPS (At post-treatment)	1/131(0.7%)	45	16	32
LT (At post-treatment)	3/131(2.2%)	52	18	-

Data are expressed as mean (± standard deviation), median (quartile 25, quartile 75) or number (proportion). Survival time are expressed as the estimated median time (95% CI)

*TIPS* Transjugular intrahepatic portosystemic shunt, *LT* Liver transplantation, *MELD* model for end-stage liver disease, *CI* confidence interval

### Factors associated with liver-related death in HH patients

We used both univariate and multivariate Cox regression analyses to identify the indicators related to liver-related death. In the univariate Cox regression analysis, the factors associated with liver-related death were male sex (HR: 1.772, 95% CI: 1.171–2.682, *P* = 0.007), combined hepatic encephalopathy (HR: 2.361, 95% CI: 1.354–4.116, *P* = 0.002), combined liver failure (HR: 1.820, 95% CI: 1.040–3.185, *P* = 0.036), combined hyponatremia (HR: 1.721, 95% CI: 1.156–2.562, *P* = 0.007), and higher TBIL level (HR: 1.004, 95% CI: 1.000- 1.007, *P* = 0.045) at baseline. In the multivariate Cox regression model, the possible variables considered were liver failure, hyponatremia, TBIL, and PT, and these four variables were further removed to see the change in HR value of the target variable (male) after the removal of a variable. After controlling for covariates that were associated with liver-related mortality in the univariate analysis, male sex (HR: 1.721, 95% CI: 1.114–2.658, *P* = 0.005), and combined hepatic encephalopathy (HR: 2.016, 95% CI: 1.101–3.693, *P* = 0.001) were found to be associated with an increase in liver-related mortality (see Table 4).

**Table 4:** Results of univariate and multivariate Cox proportional-hazards regression analysis for overall survival without transplantation

Variables	Univariate analysis		Multivariate analysis	
	HR (95% CI)	*P-*Value	HR (95% CI)	*P-*Value
Age (years)	0.993(0.977–1.009)	0.400		
Hepatitis B virus	0.995(0.667–1.483)	0.979		
Hepatitis C virus	1.165(0.650–2.090)	0.608		
Alcohol	0.920(0.551–1.537)	0.751		
Primary biliary cirrhosis	0.641(0.297–1.386)	0.258		
Male	1.772(1.171–2.682)	0.007	1.721(1.114–2.658)	0.005
Liver failure	1.820(1.040–3.185)	0.036	1.465(0.781–2.749)	0.234
Hepatic encephalopathy	2.361(1.354–4.116)	0.002	2.016(1.101–3.693)	0.001
Digestive bleeding	1.320(0.758–2.298)	0.327		
Pleural fluid infection	0.832(0.513–1.349)	0.455		
Spontaneous peritonitis	0.909(0.523–1.580)	0.736		
Hypokalemia	0.852(0.565–1.283)	0.443		
Hyponatremia	1.721(1.156–2.562)	0.007	1.429(0.916–2.229)	0.115
Hepatorenal syndrome	1.569(0.724–3.399)	0.253		
Grade III ascites	1.336(0.831–2.146)	0.232		
AST (U/L)	0.998(0.993–1.002)	0.301		
ALT (U/L)	0.998(0.993–1.003)	0.445		
GGT (U/L)	0.999(0.996–1.001)	0.265		
ALP (U/L)	0.998(0.995–1.001)	0.143		
ALB (g/L)	0.971(0.933–1.011)	0.159		
TBIL (umol/L)	1.004(1.000-1.007)	0.045	1.002(0.997–1.007)	0.379
TT (s)	1.014(0.947–1.087)	0.684		
PT (s)	1.035(0.995–1.077)	0.083	0.994(0.944–1.047)	0.825

*M* male, *F* female, *ALT* alanine aminotransferase, *AST* aspartate aminotransferase, *ALP* alkaline phosphatase, *GGT* gamma-glutamyl transpeptidase, *TBIL* total bilirubin, *ALB* albumin, *PLT* platelet, *TT* Thrombin time, *PT* prothrombin time, *HH* Hepatic Hydrothorax, *HR* hazard ratio, *CI* confidence interval

**Fig. 3:**
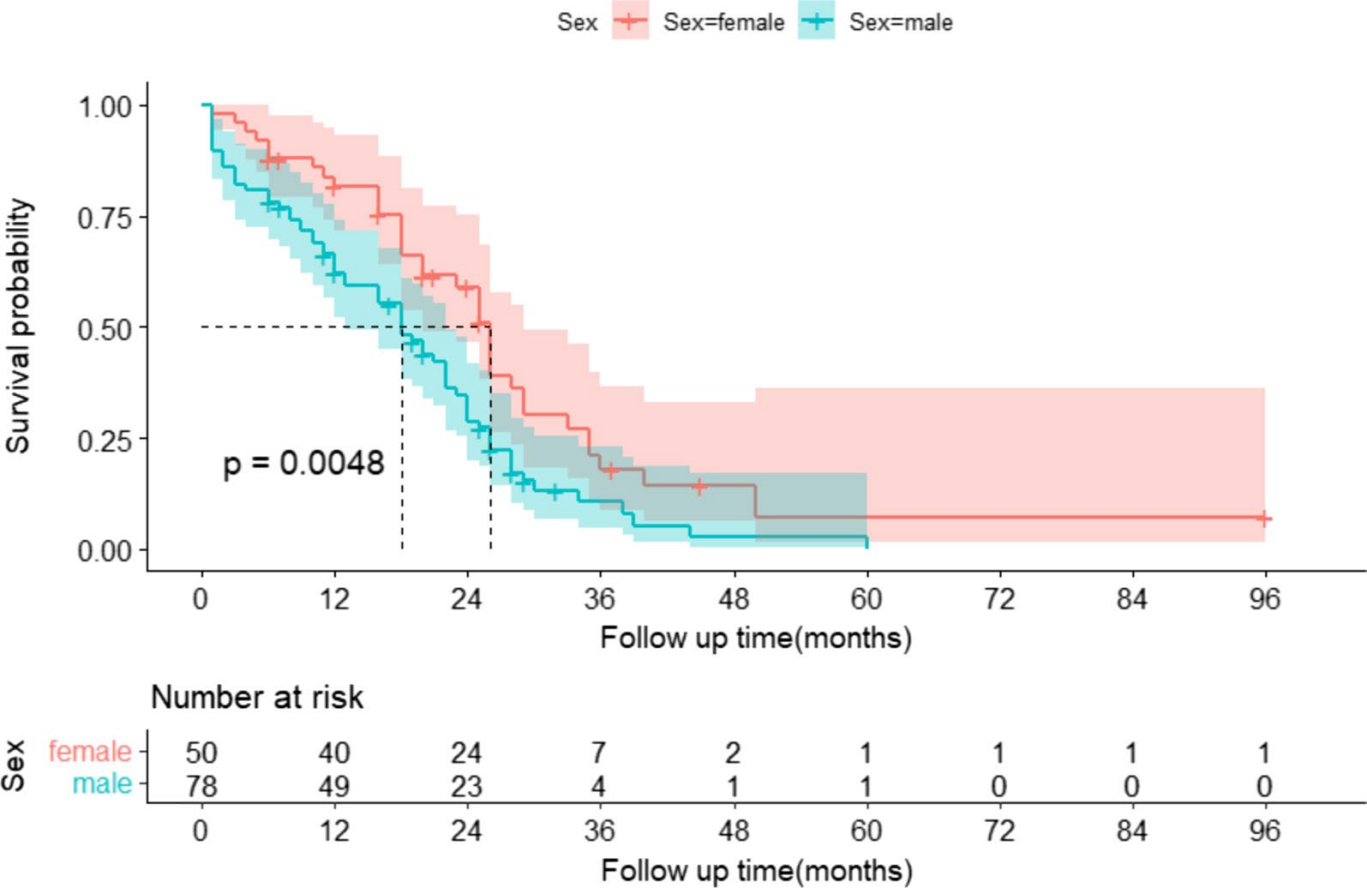
Prognostic survival by sex in HH patients without transplantation using the Kaplan–Meier method. The 95% confidence interval of the survival probability is marked by the shading. The dotted line indicates the median overall survival. The displayed *p* values follow from the log-rank test. Every tick mark indicates a censored patient

**Fig. 4:**
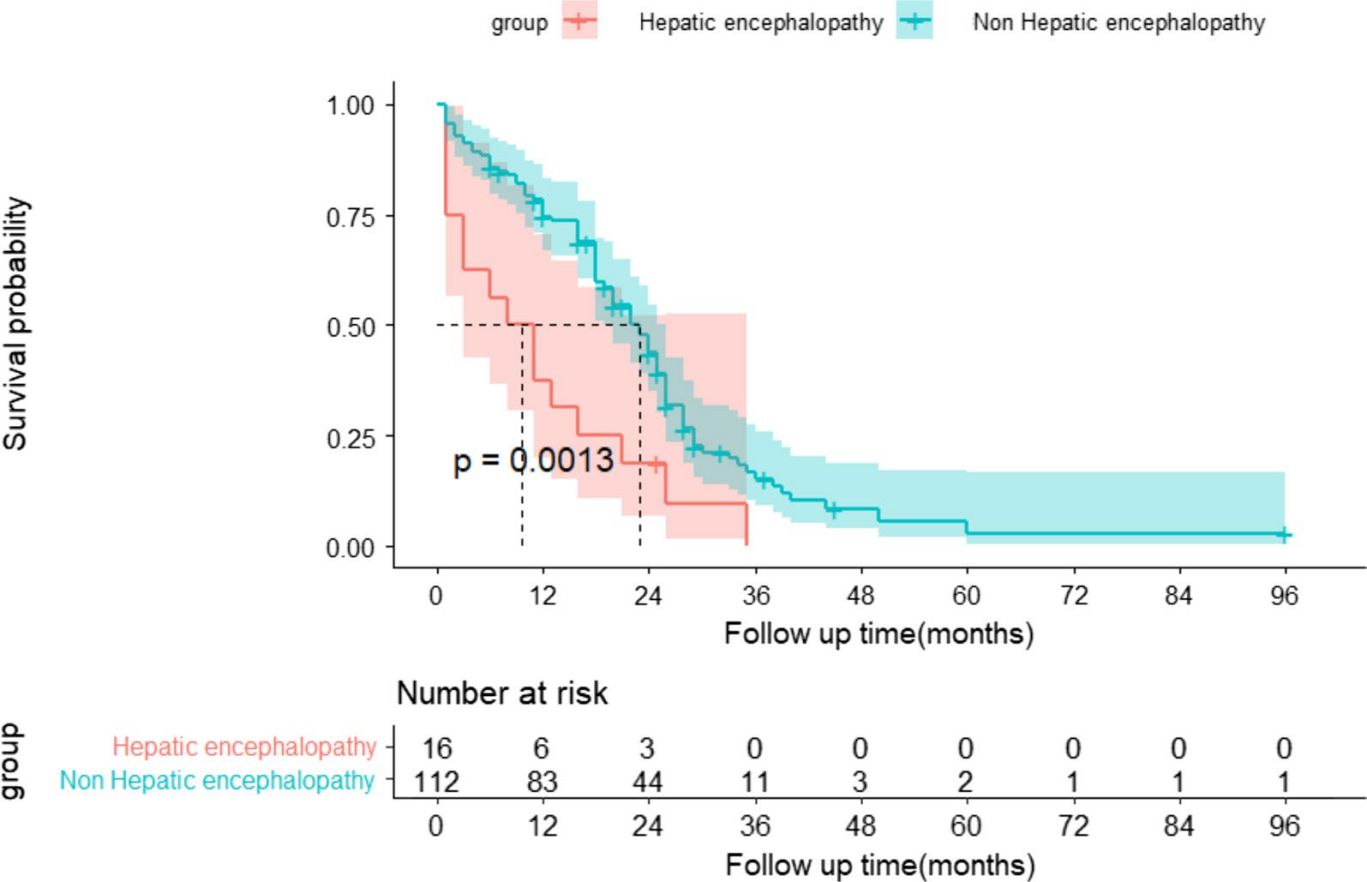
Prognostic survival of HH patients with and without hepatic encephalopathy in the absence of transplantation using the Kaplan–Meier method. The 95% confidence interval of the survival probability is marked by the shading. The dotted line indicates the median overall survival. The displayed *p* values follow from the log-rank test. Every tick mark indicates a censored patient

As depicted in Fig. 3, we compared the transplant-free survival of patients by sex using the Kaplan–Meier method. We found that the probability of transplant-free survival was significantly lower in female patients than in male patients (log-rank *P* = 0.0048). The estimated median survival time was 18 (95% CI:13–22) months for the male group and 26 (95% CI:20–29) months for the female group. As depicted in Fig. 4, we compared transplant-free survival in patients with or without combined hepatic encephalopathy using the Kaplan–Meier method. We found that patients with hepatic encephalopathy had a significantly lower probability of transplant-free survival than patients without hepatic encephalopathy (log-rank *P* = 0.0013). The estimated median survival times for patients with and without hepatic encephalopathy were 10 (95% CI:3–15) months and 23 (95% CI:19–26) months, respectively.

## Discussion

HH is a serious and difficult-to-manage complication of cirrhosis and portal hypertension that can progress to end-stage liver disease. However, the specific prognostic survival impact of HH patients and the interrelationship between the long-term impact of HH on mortality and cirrhosis-related decompensated events are currently unknown. Here, we aimed to examine the clinical characteristics and factors associated with long-term prognostic survival. More complete clinical data from hospital records of patients with decompensated cirrhosis and ascites admitted to the First Hospital of Jilin University from January 2013 to June 2021 were extracted for retrospective analysis, and factors associated with survival were further analyzed based on follow-up survival time. The diagnosis of hepatic pleural fluid was based on currently accepted clinical features of the disease, including a known diagnosis of cirrhosis, the presence of portal hypertension, the analysis of pleural effusion, and the absence of primary cardiopulmonary disease. This study is intended to further guide clinicians to clarify the clinical features of HH and the natural history of long-term survival and to seek more appropriate diagnosis and treatment.

In this study, we selected a large number of patients with decompensated cirrhosis in combination with one or both pleural effusions for a total of 131 patients with HH according to the inclusion criteria. The current study compares with the previous literature on HH, and our data represent one of the largest study cohorts [6, 13–15] with a comprehensive analysis of demographic, clinical presentation, laboratory test and examination findings data in this unique patient population.

In this study, the ratio of men to women with HH was 1.67:1; The youngest age was 25 years, the oldest was 81 years, the mean age was 52.76 ± 11.88 years and the median age was 58 years. Hepatitis B virus was the most common cause of HH, which is consistent with most studies [6, 13–15], and HH was more common in men than in women. The most common symptoms of HH were difficulty breathing and abdominal distention, which may be associated with massive pleural effusion compressing the lung tissue and massive accumulation of abdominal fluid. Splenomegaly and jaundice were the most common signs in patients with HH; These are also common signs of decompensated cirrhosis and are not clearly specific. In the thoracoabdominal imaging of HH patients, a large amount of ascites combined with a large amount of pleural fluid was commonly observed, and pleural effusion was found on the right side in approximately 87 cases (66.4%), with only a few located on the left side and bilaterally. The pathophysiology of HH is not yet fully understood [3], and the most prominent view is that pleural effusion is aided by the negative intrathoracic pressure generated during inspiration, with ascites entering the thoracic cavity directly through diaphragmatic defects of various sizes and being produced [16, 17], as the liver is anatomically similar to the diaphragm and the right diaphragm is less muscular than the left. Most patients with HH have compressive atelectasis on CT of the lungs (73.3%), which is closely related to compression of lung tissue by a large pleural effusion.

HH as a complication in patients with decompensated cirrhosis can often be combined with other complications, with peritoneal effusion being the most common, followed by electrolyte disturbances, but with hepatic encephalopathy and liver failure being the most severe and having a poor prognosis, which is in general agreement with the data reported in the literature [6].

We performed a statistical analysis of treatment modalities at the time of first diagnosis in 131 patients with HH and found that HH management was similar to portal hypertensive ascites, with restriction of sodium intake and the use of diuretics, drainage of puncture placement, and TIPS and liver transplantation as options for later treatment [3, 18–21]. Pleural catheter drainage has been previously reported [22], and no significant efficacy has been observed, but it is notable that none of our patients received this treatment. Successful treatment of patients with refractory hepatic pleural fluid who did not meet the criteria for TIPS by repairing the diaphragmatic defect with televised thoracoscopic surgery (VATS) has also been reported in the literature [23], but none of our patients attempted this treatment. Among our patients, 20 (15%) were treated with drugs alone, and 111 (75%) were treated with drugs combined with thoracic intubation, which suggests that most pleural effusions were massive and required puncture and drainage to relieve the compression of lung tissue and thereby relieve the symptoms of difficulty in breathing. There was no statistically significant difference in MELD scores or survival time between patients first diagnosed with drugs alone and those with drugs combined with thoracic intubation. Only one patient in our study was found to have undergone TIPS at a later stage of treatment, and three patients finally underwent liver transplantation. The three patients who underwent liver transplantation remain alive today.

By following the survival time of patients, we found that the overall prognosis of HH patients was poor, with approximately more than half dying within one year of the onset of symptoms and only 34 cases (33.7%) surviving after one year. Patients survived significantly longer after receiving liver transplantation, considering that liver transplantation may be a better treatment option than drugs and chest tube drainage; However, caution is needed in interpreting these data as only a very small number of patients, 2.2% of the entire study population, received liver transplantation. Furthermore, it is possible that patients with a better prognosis were more likely to opt for liver transplantation treatment. In addition, the selection of liver transplant recipients is extremely complex, and although patients with advanced liver failure are selected based on screening criteria, transplant candidates are rarely comorbid with other diseases. Therefore, retrospective conclusions based on a very small amount of data may be inaccurate.

We also found that male sex and hepatic encephalopathy were associated with an increase in liver-related mortality. In a recently published retrospective cohort study, a MELD-Na score ≥ 16, ALBI grade III, hepatorenal syndrome, or severe ascites delineated high-mortality risk groups [6]. Our conclusions differ because we excluded the influence of scoring criteria such as the MELD-Na score, MELD score, and ALBI score, considering that these contain different independent variables, making it difficult to analyze the most realistic influences more intuitively. In patients with HH, early identification of patients with a poor prognosis based on factors affecting the prognosis, together with targeted treatment, is particularly important. At the same time, some comorbidities as diabetes, hypertension, dyslipidemia, and cardiovascular disease might be associated with survival, but considering that we are exploring prognostic factors related to liver-related mortality or survival, we did not include these variables further in the analysis.

There are several limitations of our study. First, this study is a single-center retrospective analysis and does not exclude the influence of subjective factors by patients and medical record keepers. Second, taking into account changes in compliance with sodium restriction and diuretic dose throughout follow-up made data collection relatively difficult. Therefore, throughout the natural history of HH, we did not specify the impact of various treatment modalities on the patient prognosis, which can certainly be an important prognostic modifier, nor did we specify the impact of clinical presentations related to HH on the patient prognosis, but this may help better understand the impact of decompensated events on the prognosis of patients with HH. Third, the analysis performed to identify potential risk factors is merely exploratory and our findings are subject to random high bias. Fourth, the small number of patients undergoing TIPS and liver transplantation did not allow inclusion in the Cox proportional hazards model survival analysis. Fifth, variables that did not satisfy the proportional hazard assumption were not assessed as associated with survival time, thus ignoring variables associated with potentially meaningful outcomes, while some of the associations we found may be driven by these neglected variables.

In summary, our findings suggest that male sex and hepatic encephalopathy are predictive factors of poor prognosis in HH patients. Confirmation is needed by properly designed prospective studies.

## Data Availability

The datasets used and/or analyzed during the current study are available from the corresponding author upon reasonable request.

## Abbreviations

M: Male
F: Female
ALT: Alanine aminotransferase
AST: Aspartate aminotransferase
ALP: Alkaline phosphatase
GGT: Gamma-glutamyl transpeptidase
CHE: Cholinesterase
ALB: Albumin
TBIL: Total bilirubin
DBIL: Direct bilirubin
IBIL: Indirect bilirubin
Cr: Creatinine
BUN: Blood urea nitrogen
FBG: Fasting blood glucose
NE: Neutrophil absolute count
LY: Lymphocyte absolute count
NLR: Neutrophil-to-lymphocyte ratio
PLT: Platelet
TT: Thrombin time
APTT: Activated partial thromboplastin time
INR: International normalized ratio
PT: Prothrombin time
PTA: Prothrombin activity
TIPS: Transjugular intrahepatic portosystemic shunt
LT: Liver transplantation
MELD: Model for end-stage liver disease
ALBI: Albumin-bilirubin
HH: Hepatic Hydrothorax
HR: Hazard ratio
CI: Confidence interval
